# Smoking is associated with the incidence of AMS: a large-sample cohort study

**DOI:** 10.1186/2054-9369-1-16

**Published:** 2014-07-23

**Authors:** Pan Song, Ji-hang Zhang, Jun Qin, Xu-bin Gao, Jie Yu, Xu-gang Tang, Cai-fa Tang, Lan Huang

**Affiliations:** Institute of Cardiovascular Diseases of PLA, Xinqiao Hospital, Third Military Medical University, Chongqing, 400037 People’s Republic of China; Institute of Internal Medicine-Neurology of PLA, Xinqiao Hospital, Third Military Medical University, Chongqing, 400037 People’s Republic of China

**Keywords:** High altitude, Acute mountain sickness, Smoking

## Abstract

**Background:**

In recent years, the number of people visiting high altitudes has increased. After rapidly ascending to a high altitude, some of these individuals, who reside on plains or other areas of low altitude, have suffered from acute mountain sickness (AMS). Smoking interferes with the body's oxygen metabolism, but research about the relationship between smoking and AMS has yielded controversial results.

**Methods:**

We collected demographic data, conducted a smoking history and performed physical examinations on 2000 potential study participants, at sea level. Blood pressure (BP) and pulse oxygen saturation (SpO_2_) were measured for only some of the patients due to time and manpower limitations. We ultimately recruited 520 smokers and 450 nonsmokers according to the inclusion and exclusion criteria of our study. Following acute high-altitude exposure, we examined their Lake Louise Symptom (LLS) scores, BP, HR and SpO_2_; however, cerebral blood flow (CBF) was measured for only some of the subjects due to limited time, manpower and equipment.

**Results:**

Both the incidence of AMS and Lake Louise Symptom (LLS) scores were lower in smokers than in nonsmokers. Comparing AMS-related symptoms between nonsmokers and smokers, the incidence and severity of headaches and the incidence of sleep difficulties were lower in smokers than in nonsmokers. The incidences of both cough and mental status change were higher in smokers than in nonsmokers; blood pressure, HR and cerebral blood flow velocity were lower in smokers than in nonsmokers.

**Conclusion:**

Our findings suggest that the incidence of AMS is lower in the smoking group, possibly related to a retardation of cerebral blood flow and a relief of AMS-related symptoms, such as headache.

## Background

In recent years, mountain climbing and trekking at high altitudes have become increasingly popular recreational activities. After rapidly ascending to high altitudes, some plains residents suffer from a clinical syndrome associated with a series of symptoms, known as acute mountain sickness (AMS). Low-barometric hypoxia at high altitudes is the fundamental cause of AMS. Smoking interferes with the body's oxygen metabolism. To date, researchers have studied the relationship between smoking and AMS but have reported controversial results. Some researchers found that smoking was not related to AMS susceptibility [1–3]. However, Hultgren [4] concluded that smoking increased the incidence of hypoxia, resulting in susceptibility to AMS. Additionally, other studies found that smoking reduced the incidence of AMS. To further study the relationship between smoking and AMS, we designed this cohort study [5, 6].

## Methods

### Subjects

Eligible participants for this study had to be healthy plains residents and meet our definitions of smoking and nonsmoking. A smoker was defined as an individual who smoked 10 or more cigarettes per day for at least 6 months. Nonsmokers were defined as never having smoked cigarettes. Participants with any one of the following conditions were excluded: Tibetan residency, long-term high-altitude living history (>3,000 m for six months or more), high-altitude exposure history (>3,000 m) during the past six months, occasional smoking, or poor health. Each subject was fully informed and volunteered to participate in this study, and all subjects signed an informed consent. They were allowed to quit the study at any time without providing a reason. This study was approved by the Ethics Committee of Xinqiao Hospital of the Second Clinic Medical College of Third Military Medical University.

We collected demographic data, conducted a smoking history and performed physical examinations on 2,000 potential study participants, at sea level. Blood pressure (BP) and pulse oxygen saturation (SpO_2_) were measured for only some of the participants due to time and manpower limitations. We ultimately recruited 520 smokers and 450 nonsmokers according to the inclusion and exclusion criteria of our study. Following acute high-altitude exposure, we examined their LLS scores, BP, HR and SpO_2_; however, cerebral blood flow (CBF) was measured for only some of the subjects due to limited time, manpower and equipment.

Eleven subjects did not ascend to high altitude for personal reasons. Nine subjects were removed from the study due to incomplete information. The data of an additional 8 subjects who ascended to high altitude were not collected.

The age, height and weight of each recruited subject were collected at sea level. The BP, HR and SpO_2_ of 838 subjects (373 smokers and 465 nonsmokers) were measured at sea level due to limited time and manpower. Following high-altitude exposure, 506 LLS scores, 503 BP, HR and SpO_2_ readings, and 225 CBF measurements, were collected from the smokers, and 436 LLS scores, 428 BP, HR and SpO_2_ readings, and 130 CBF measurements, were collected from the nonsmokers. All subjects were healthy individuals without any history of cardiopulmonary disease.

### Trek log

Baseline data were collected at Chengdu (500 m). The subjects then traveled to Lhasa (3,700 m) from Chengdu by air within two hours. Data were collected at high altitude 24 h after initial high-altitude exposure.

### Examinations

The following demographic data were collected during recruitment: gender, age, height and weight. An epidemiology questionnaire about AMS was used to record information about each patient’s symptoms and signs of AMS. Symptoms included headache, dizziness, lightheadedness, gastrointestinal symptoms, sleep difficulty, fatigue, weakness, tightness in the chest, palpitations, shortness of breath, constipation, abdominal distension, diarrhea, tinnitus, vertigo and decreased activity. We diagnosed AMS using the Lewis Lake International Diagnostic criteria (LLS) [7], which included the following 5 symptoms: headache, dizziness, gastrointestinal symptoms, sleep problems and fatigue. AMS was defined as a total score of 3 or more symptoms in addition to headache. Arterial SpO_2_, heart rate (Nonin Onyx® 9550, Nonin Medical, Inc., USA) and arterial blood pressure (OMRON HEM-6200, OMRON healthcare Ltd., Japan) were measured following rest in a seated position for at least 30 min. Blood flow velocity of the middle cerebral artery (MCAv) was also measured. MCAv was estimated via continuous measurements of backscattered Doppler signals from the right middle cerebral artery using a 2 MHz pulsed Doppler ultrasound system (EME TC2021-III, NICOLET, USA).

### Statistical methods

SPSS 13.0 was used for data analyses. An independent sample *t* test was used to analyze the differences in data between smokers and nonsmokers, which included demographic data, vital signs and cerebral blood flow velocity. Chi-square tests were utilized to compare differences in the incidence of AMS and each symptom between the two groups. The differences in LLS scores and symptom scores between the two groups were compared by non-parametric tests.

## Results

### AMS and symptoms

**Figure 1:**
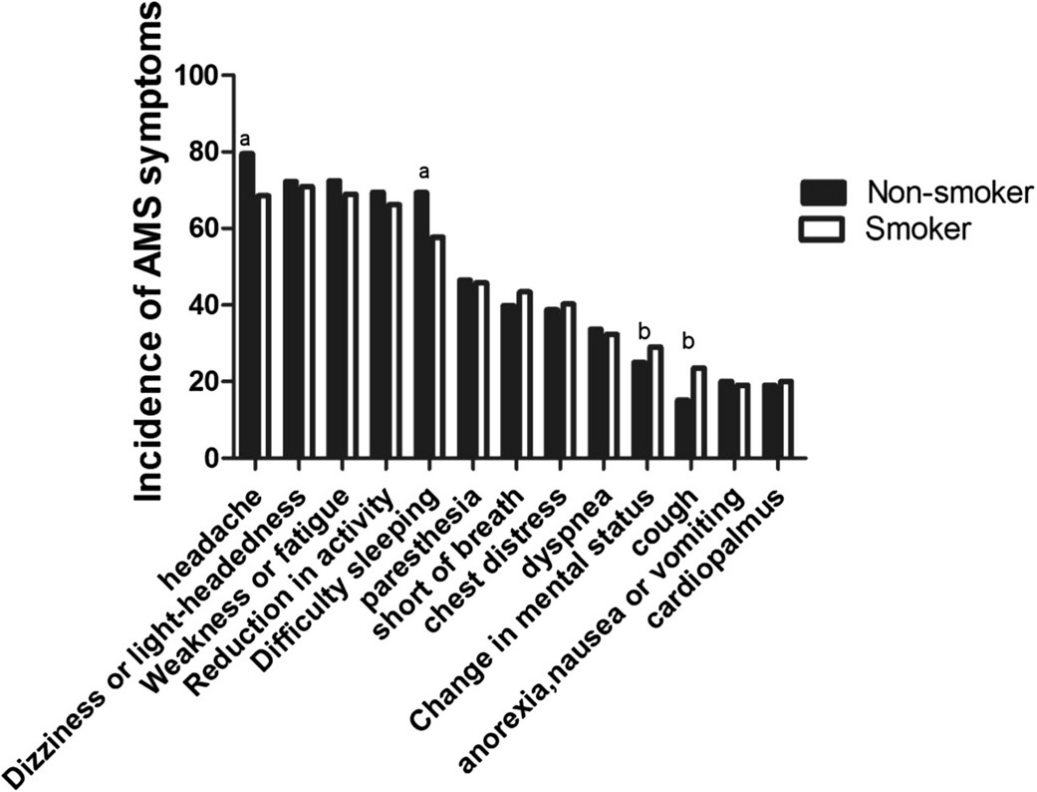
**Comparison of the incidence of symptoms between the two groups. a** represents the incidence of symptoms of non-smokers being higher than the incidence of symptoms in smokers: **b** represents the incidence of symptoms of non-smokers being lower than the incidence of symptoms in smokers.

There were no significant differences in age, height or weight between the smokers and nonsmokers (Table 1). The incidence of AMS in the nonsmokers was 66.53%: the incidence of AMS in the smokers was 56.58%, and a significant difference existed between the two groups (*P* < 0.05). The LLS score of the smokers (2.86 ± 2.46) was lower than that of the nonsmokers (3.46 ± 2.49). Regarding the five symptoms of AMS, there were significant differences in headache and sleep difficulty scores between the two groups, whereas the scores and incidences of dizziness, lightheadedness, gastrointestinal symptoms and fatigue or weakness revealed no significant differences (Table 2). The incidences of cough (24%) and mental status changes (29%) were higher in smokers than in nonsmokers (15% and 25%, respectively), but the incidences of headache (69%) and sleep difficulty (58%) were lower in smokers than in nonsmokers (79% and 69%, respectively). There were no statistically significant differences in either the incidence or severity of other symptoms between the two groups (Figure 1).

**Table 1:** **Comparison of age, height and weight**

Index	Non-smoker (*n* = 436)	Smoker (*n* = 506)
Age (year)	23.61 ± 4.31	23.73 ± 3.49
Height (cm)	171.30 ± 4.42	171.64 ± 4.93
Weight (kg)	64.00 ± 6.75	64.07 ± 7.44

**Table 2:** **Comparison of LLS and symptom scores**

Index	Non-smoker (*n* = 436)	Smoker (*n* = 506)
LLS score	3.78 ± 2.17	3.34 ± 2.09^a^
Headache	0.96 ± 0.61	0.81 ± 0.65^a^
Dizziness/lightheadedness	0.82 ± 0.60	0.77 ± 0.56
Weakness/fatigue	0.81 ± 0.58	0.76 ± 0.58
Anorexia, nausea or vomiting	0.22 ± 0.46	0.20 ± 0.42
Difficulty sleeping	0.99 ± 0.83	0.80 ± 0.81^a^

^a^ *P* < 0.05 compared with non-smokers.

### Blood pressure, HR, SpO_2_

At sea level, the systolic blood pressure (SBP), diastolic blood pressure (DBP), heart rate (HR), and pulse oxygen saturation (SpO_2_) of the smoking group did not differ from those of the nonsmoking group (Table 3). At high altitude, the SBP (118.3 ± 11.77), DBP (78.04 ± 9.54), and HR (83.38 ± 11.98) of the smokers were lower than those of the nonsmokers (120.29 ± 11.90, 80.13 ± 10.17 and 85.16 ± 12.34, respectively), and only SpO_2_ exhibited no difference between the two groups (Table 3).

**Table 3:** **Comparison of BP, HR, SpO**_**2**_ **at 500 m and high altitude**

Index	500 m		High altitude	
	Non-smoker (*n* = 373)	Smoker (*n* = 465)	Non-smoker (*n* = 428)	Smoker (*n* = 503)
SBP	115.76 ± 11.61	115.0 ± 10.60	120.29 ± 11.90	118.3 ± 11.77^a^
DBP	72.87 ± 9.67	72.29 ± 8.93	80.13 ± 10.17	78.04 ± 9.54^a^
HR	66.45 ± 10.48	65.40 ± 10.42	85.16 ± 12.34	83.38 ± 11.98^a^
SpO_2_	98.07 ± 0.99	98.10 ± 1.05	88.70 ± 3.25	88.87 ± 3.13

^a^ *P* < 0.05 compared with non-smokers.

### Cerebral blood flow velocity

At high altitude, the systolic Cerebral blood flow velocity (SCBF, 101.12 ± 14.27), diastolic Cerebral blood flow velocity (DCBF, 47.04 ± 8.73) and average Cerebral blood flow velocity (MCBF, 66.43 ± 11.04) of the smoking group were each significantly lower than the corresponding variables of the nonsmoking group (105.98 ± 17.06, 49.78 ± 10.19 and 69.64 ± 12.96, respectively, Table 4).

**Table 4:** **Comparison of cerebral blood flow velocities at high altitude**

Index	Non-smoker (*n* = 130)	Smoker (*n* = 225)
Systolic velocity	105.98 ± 17.06	101.12 ± 14.27^a^
Diastolic velocity	49.78 ± 10.19	47.04 ± 8.73^a^
Mean velocity	69.64 ± 12.96	66.43 ± 11.04^a^

^a^ *P* < 0.05 compared with non-smokers. Due to technical problems, only a subset of subjects (*n* = 355) underwent cerebral blood flow velocity tests.

## Discussion

We found that the incidence of AMS of the smoking group decreased by 10% compared with that of the nonsmoking group. This result contrasts with previous studies on AMS risk factors in tourists and climbers [1–3] but confirms a tendency noted in another prospective cohort study (crude *OR* 0.66, 95% CI 0.41-1.07, *P* = 0.09) [8]. Our results are consistent with the results of Wu *et al.* [6], who reported an 11% decrease in the incidence of AMS in smokers compared with nonsmokers. Hultgren [4] believes that smoking may increase the incidence of AMS and is not conducive to high altitude acclimatization because a larger amount of CO hemoglobin in smokers’ blood will increase the likelihood of hypoxia. Although the incidence of AMS in the smoking population was lower than that in the nonsmoking population, the incidences of mental status changes and cough in smokers at high altitude were 4% and 7% higher than nonsmokers under the same conditions.

Our results showed slower cerebral blood flow velocities in smokers, as well as a lower incidence of headaches. This result may explain why AMS incidence is lower in smokers. Baumgartner RW reported that the occurrence of AMS was related to cerebral blood flow [9]. The primary factors affecting cerebral blood flow are cerebral arteriovenous pressure differences and cerebral blood flow resistance. Under normal circumstances, internal jugular venous pressure is similar to right atrial pressure and remains relatively unchanged; therefore, the primary factor affecting cerebral blood flow is arterial pressure. Because of the autoregulation of cerebral blood flow, fluctuations in blood pressure do not cause fluctuations in cerebral blood flow under normal circumstances. However, exposure to high altitude damages cerebral blood flow autoregulation [10], resulting in an increased susceptibility of cerebral blood flow to changes in blood pressure. We believe that the cerebral blood flow of smokers is lower than that of nonsmokers, not as a result of superior autoregulation of cerebral blood flow at high altitude but because of lower BP in this population. However, we did not research the underlying mechanisms of these findings.

Another possible explanation for our findings may be related to superior sleep quality in smokers. Sojourners to high altitudes frequently experience sleep disturbances, often reporting restlessness and sleeplessness at night. Some may describe a feeling of suffocation upon awakening from sleep. Szymczak *et al*. [11] used the Pittsburgh Sleep Quality Index (PSQI) to survey subjective sleep quality in volunteers who ascended rapidly from plains to a 6119-m altitude. He found that volunteers’ scores were significantly increased at high altitude (2.88 ± 1.70 at plain *vs*. 7.58 ± 4.82 at high altitude). Up to 53% of the volunteers complained of decreased sleep quality due primarily to prolonged periods of time before falling asleep, as well as decreased sleep efficiency and damaged sleep continuity. The main factors that affected sleep were frequent awakenings, low temperatures and breathing difficulties [12]. In addition to changes noted in subjective sleep quality, sleep structure is also affected by high altitude [13, 14]. A characteristic waxing and waning breathing pattern, known as periodic breathing, accompanies sleep and leads to difficulty sleeping at high altitudes: it should be noted that periodic breathing at high altitudes is different from the typical waxing and waning in tidal volumes observed with periodic breathing in the setting of heart failure or in the somewhat chaotic and irregular occurrences of apnea associated with opiate use. Research shows that the hypoxic ventilatory response to faster breathing, an increase in carbon dioxide emissions due to hypocapnia that causes respiratory modulation instability, is the mechanism of periodic breathing. Nicotine and carbon monoxide affect the regulation of breathing [15, 16]. The reason why smokers sleep better may be that high concentrations of nicotine and carbon monoxide reduce breathing pattern instability, subsequently reducing the frequency of awakenings during sleep.

### Innovations and limitations

The primary innovation of our study is the inclusion of 436 nonsmokers and 506 smokers, allowing for the identification of small differences between the groups that were previously unknown. The subjects in this study were young males aged 16–22 years old; therefore, the effects of smoking were not completely examined.

We did not measure either CO or NO levels in exhaled air or blood, nor did we measure COHb levels or determine the relationships of any of these parameters to AMS symptom scores. Sleep quality, which is measured with actimetry, as well as the quantification of ventilatory responses to hypoxia and hypercapnia, may have provided further insight into these relationships. As smoking reportedly reduces pain perception, we could not fully exclude the possibility that the perception of AMS symptoms may be less in smokers compared with nonsmokers, which may also explain the lower incidence of AMS in smokers.

## Conclusion

Our findings suggest that the incidence of AMS is lower in the smoking group, possibly related to a retardation of cerebral blood flow and a relief of AMS-related symptoms, such as headache.

## Abbreviations

AMS: Acute mountain sickness
CO: Carbon monoxide
NO: Nitric oxide
BP: Blood pressure
SBP: Systolic blood pressure
DBP: Diastolic blood pressure
HR: Heart rate
SpO2: Pulse oxygen saturation
MCAv: Blood flow velocity in the middle cerebral artery
LLS: Lake Louise symptom
SCBF: Systolic cerebral blood flow velocity
DCBF: Diastolic cerebral blood flow velocity
MCBF: Mean cerebral blood flow velocity
MHz: Megahertz
USA: United States of America.

## References

[1] Gaillard S, Dellasanta P, Loutan L, Kayser B (2004). Awareness, prevalence, medication use, and risk factors of acute mountain sickness in tourists trekking around the Annapurnas in Nepal: a 12-year follow-up. High Alt Med Biol.

[2] Schneider M, Bernasch D, Weymann J, Holle R, Bartsch P (2002). Acute mountain sickness: influence of susceptibility, preexposure, and ascent rate. Med Sci Sports Exerc.

[3] Beidleman BA, Tighiouart H, Schmid CH, Fulco CS, Muza SR (2013). Predictive models of acute mountain sickness after rapid ascent to various altitudes. Med Sci Sports Exerc.

[4] Hultgren HN (1997). High altitude medicine.

[5] MacLean N (1979). Smoking and acclimatisation to altitude. Br Med J.

[6] Wu TY, Ding SQ, Liu JL, Jia JH, Chai ZC, Dai RC, Zhao JZ, Tang QD, Kayser B (2012). Smoking, acute mountain sickness and altitude acclimatisation: a cohort study. Thorax.

[7] Roach RC BP, Hackett PH, Oelz O, Sutton JR, Houston CS, Coates G, Lake Louise AMS Scoring and C. Committee (1993). **The Lake Louise acute mountain sickness scoring system**. Hypoxia and molecular medicine: proceedings of the 8th International Hypoxia Symposium, Lake Louise, Canada.

[8] Richalet JP, Larmignat P, Poitrine E, Letournel M, Canouï-Poitrine F (2012). Physiological risk factors of severe high altitude illness: a prospective cohort study. Am J Respir Crit Care Med.

[9] Baumgartner RW, Bärtsch P, Maggiorini M, Waber U, Oelz O (1994). Enhanced cerebral blood flow in acute mountain sickness. Aviat Space Environ Med.

[10] Ainslie PN, Burgess K, Subedi P, Burgess KR (2007). Alterations in cerebral dynamics at high altitude following partial acclimatization in humans: wakefulness and sleep. J Appl Physiol.

[11] Szymczak RK, Sitek EJ, Sławek JW, Basiński A, Siemiński M, Wieczorek D (2009). Subjective sleep quality aherations at high altitude. Wilderness Environ Med.

[12] Bloch KE, Latshang TD, Turk AJ, Hess T, Hefti U, Merz TM, Bosch MM, Barthelmes D, Hefti JP, Maggiorini M, Schoch OD (2010). Nocturnal periodic breathing during acclimatization at very high altitude at Mount Muztagh Ata (7,546 m). Am J Resp Crit Care.

[13] Reite M, Jackson D, Cahoon RL, Weil JV (1975). Sleep physiology at high altitude. Electroencephalogr Clin Neurophysiol.

[14] Johnson PL, Edwards N, Burgess KR, Sullivan CE (2010). Sleep architecture changes during a trek from 1400 to 5000 m in the Nepal Himalaya. J Sleep Res.

[15] Prabhakar NR (1999). NO and CO as second messengers in oxygen sensing in the carotid body. Respir Physiol.

[16] Argacha JF, Xhaët O, Gujic M, Adamopoulos D, Beloka S, Dreyfuss C, Degaute JP, van de Borne P (2008). Nicotine increases chemoreflex sensitivity to hypoxia in nonsmokers. J Hypertens.

